# Dual Fluorescent Reporter Pig for Cre Recombination: Transgene Placement at the *ROSA26* Locus

**DOI:** 10.1371/journal.pone.0102455

**Published:** 2014-07-15

**Authors:** Shun Li, Tatiana Flisikowska, Mayuko Kurome, Valeri Zakhartchenko, Barbara Kessler, Dieter Saur, Alexander Kind, Eckhard Wolf, Krzysztof Flisikowski, Angelika Schnieke

**Affiliations:** 1 Chair of Livestock Biotechnology, Technische Universität München, Freising, Germany; 2 Molecular Animal Breeding and Biotechnology, Ludwig-Maximilians-Universität München, Oberschleissheim, Germany; 3 Klinikum Rechts der Isar II, Technische Universität München, Munich, Germany; Ohio State University Comprehensive Cancer Center, United States of America

## Abstract

We are extending the Cre/loxP site-specific recombination system to pigs, focussing on conditional and tissue-specific expression of oncogenic mutations to model human cancers. Identifying the location, pattern and extent of Cre recombination in vivo is an important aspect of this technology. Here we report pigs with a dual fluorochrome cassette under the control of the strong CAG promoter that switches expression after Cre-recombination, from membrane-targeted tandem dimer Tomato to membrane-targeted green fluorescent protein. The reporter cassette was placed at the porcine *ROSA26* locus by conventional gene targeting using primary mesenchymal stem cells, and animals generated by nuclear transfer. Gene targeting efficiency was high, and analysis of foetal organs and primary cells indicated that the reporter is highly expressed and functional. Cre reporter pigs will provide a multipurpose indicator of Cre recombinase activity, an important new tool for the rapidly expanding field of porcine genetic modification.

## Introduction

Site-specific recombination systems such as Cre/loxP are powerful and versatile tools for mouse experimental genetics that enable precisely controlled conditional gene expression and many other modifications in whole animals [1]. We are particularly interested in conditional and tissue-specific expression of oncogenic mutations to model human cancers [2], [3]. In mice, Cre reporter strains provide a means of monitoring the location, pattern and extent of Cre recombination *in vivo.* A very successful and reliable reporter has been developed using a highly expressed dual fluorochrome cassette that switches expression after Cre-recombination, from membrane-targeted tandem dimer Tomato (mTomato) to membrane-targeted green fluorescent protein (mGFP), placed at the *Rosa26* locus for ubiquitous expression [4]. This is currently the ‘gold standard’ Cre reporter system, because it provides sensitive real-time visualisation capable of identifying even single green Cre-recombined cells within a background of red non-recombined cells [5] and conversely a few red non-recombined cells in a background of green Cre-recombined cells [6].

Here we report pigs with an mTomato, mGFP dual fluorescent Cre reporter under the control of the CAG promoter placed by gene targeting at the porcine *ROSA26* locus to ensure ubiquitous expression. These animals provide a multipurpose indicator of Cre recombinase activity, an important new tool for the rapidly expanding field of porcine genetic modification.

## Materials and Methods

Animal experiments were approved by the Government of Upper Bavaria (permit number 55.2-1-54-2532-34-09) and performed according to the German Animal Welfare Act and European Union Normative for Care and Use of Experimental Animals.

### 3′RACE (3′ rapid amplification of cDNA ends) analysis of porcine *ROSA26*

3′RACE analysis was carried out using the FirstChoice® RLM-RACE kit (Ambion) according to the manufacturer’s protocol. Primers used were: Ex1F1 (5′ CGCCTAGAGAAGAGGCTGTG 3′), which hybridises to porcine *ROSA26* exon 1; and nest primer Ex1F2 (5′ AGAAGAGGCTGTGCTCTGG 3′), which also hybridises to exon 1. Thermal cycling parameters were: 30 sec, 98°C; then 35 cycles of: 5 sec, 98°C; 5 sec, 63°C; 15 sec, 72°C; followed by 1 min, 72°C. The size of the amplified product was 800 bp.

### Porcine *ROSA26* gene targeting vectors GCROSA and TGROSA

The DNA sequence on porcine chromosome 13 (NCBI accession number NW_003611693) was used to generate the promoter trap gene targeting vector GCROSA and TGROSA. GCROSA comprised: a 2.166 kb 5′ short arm of homology corresponding to a region of *ROSA26* intron 1 from position 31986 to 34149 (NW_003611693); a 159 bp adenoviral splice acceptor; a 7.739 kb floxed β-Geo caste; a 1.681 kb mCherry-poly A cassette; a 4.675 kb 3′ homology long arm. The 7.739 kb floxed β-geo cassette of targeting vector comprised: a 34 bp loxP site; a 3.707 kb promoterless β-Geo cassette; three polyadenylation signals derived from SV40, bovine growth hormone and cytomegalovirus (CMV); a 3.053 kb HPRT stuffer sequence; a second loxP site. TGROSA comprised: a 2.110 kb 5′ short arm of homology corresponding to a region of *ROSA26* intron 1 from position 32043 to 34152 (NW_003611693); a 159 bp adenoviral splice acceptor; a 426 bp promoterless blasticidin resistance gene (*bsr*) followed by two polyadenylation signals derived from SV40 and the bovine growth hormone gene; a 1.715 kb CAG promoter (chicken beta-actin promoter with CMV enhancer); a 2.440 kb membrane-targeted tdTomato (mTomato) gene flanked by two loxP sites; a 1.099 kb membrane-targeted EGFP (mEGFP) gene; and a 4.647 kb 3′ long arm of homology. The mTomato, mEGFP cassette was derived from Addgene plasmid 17787.

### Generation of gene targeted porcine mesenchymal stem cell (MSC) clones

MSCs were isolated by standard methods from bone marrow or subcutaneous fat of a 6-month old male German Landrace pig. MSCs were cultured in advanced Dulbecco’s modified Eagle medium (DMEM) (Gibco), 2 mM GlutaMAX, 1× non-essential amino acids, 10% foetal calf serum (FCS gold, lot no. A15109-2859), 5 ng/ml FGF-2 (PromoKine) at 37°C, 5% CO_2_ and passaged using Accutase (PAA). Samples of 1×10^6^ MSCs were electroporated with 10 µg GCROSA or TGROSA vector DNA linearised with *NotI* or *Mlu*I, and 500 µg/ml G418 or 8 µg/ml blasticidin (Invivogen) selection applied 2 days later. Individual stable transfected cell clones were isolated, samples of each clone cryopreserved at an early stage and replicate samples cultured further for DNA and RNA analyses.

### PCR and RT-PCR analysis of targeted MSC clones

Targeted cell clones were identified by PCR using primer targF (5′ TCTGCTGCCTCCTTTTCCTA 3′), which hybridises to a point in porcine *ROSA26* intron 1 outside the 5′ homologous arm of the targeting vector, and primer targSAR (5′ GAAAGACCGCGAAGAGTTTG 3′), which hybridises to the adenoviral splice acceptor. PCR was carried out using the 5 PRIME Extender System (5 PRIME GmbH). Thermal cycling parameters were: 2 min, 94°C; then 38 cycles of: 20 sec, 94°C; 20 sec, 65°C; 2.5 min, 72°C; followed by 5 min, 72°C. The size of the diagnostic amplified product was 2.630 kb.

Long-range PCR across the 3′ junction of the targeted *ROSA26* allele was carried out using either forward primer mCF (5′ CCTGTCCCCTCAGTTCATGT 3′) for mCherry, or mTF (5′ ACATGGCCGTCATCAAAGAG 3′) for mTomato, in combination with reverse primer LR (5′ CTTGCCCCACGACAAGATCA 3′), which hybridises to a point in porcine *ROSA26* intron 2 outside the 3′ homologous arm of either targeting vector. PCR was carried out using the 5 PRIME Extender System (5 PRIME GmbH). Thermal cycling parameters were: 3 min, 93°C; then 38 cycles of: 15 sec, 93°C; 30 sec, 62°C; 7 min, 68°C; followed by 5 min, 68°C. The size of the amplified product was 6.331 kb (GCROSA) or 7.868 kb (TGROSA).

The wild type *ROSA26* allele was amplified using primer targF (sequence above) and endoR (5′ GTTTGCACAGGAAACCCAAG 3′) using the 5 PRIME Extender System. Thermal cycling parameters were: 2 min, 94°C; then 38 cycles of: 20 sec, 94°C; 20 sec, 60°C; 3 min, 72°C; followed by 5 min, 72°C. The diagnostic amplified product was 3.2 kb.

Expression of *bsr* mRNA directed by the *ROSA26* promoter was detected by amplification from exon 1 to the *bsr* cassette by two-step RT-PCR with M-MuLV transcriptase (New England Biolabs) according to the manufacturer’s instructions. Primers used were Ex1F1 (sequence above) and reverse primer bsR (5′ AGCAATTCACGAATCCCAAC 3′), which hybridises to the *bsr* cassette. Thermal cycling parameters were: 2 min, 95°C; then 38 cycles of: 30 sec, 95°C; 30 sec, 60°C; 1 min, 72°C; followed by 5 min, 72°C. The diagnostic amplified product was 500 bp.

Expression of mTomato RNA transcribed from the CAG promoter was detected by two-step RT-PCR using primers mTF (sequence above) and mTR (5′ GTACAGCTCGTCCATGCCGTA 3′), which hybridises to the mTomato gene. Thermal cycling parameters were as above. The diagnostic amplified product was 686 bp.

Expression of wild-type *ROSA26* mRNA was detected by two-step RT-PCR using primers Ex1F1 (sequence above) and Ex2R (5′ TATGCTTAGCAGCTTCCTC 3′), which hybridises to *ROSA26* exon 2, and primers Ex1F2 (sequence above) and Ex4R (5′ CTGCTGTGGCTGTGGTGTAG 3′) which hybridises to *ROSA26* exon 4, using Phire Hot Start II DNA Polymerase (Thermo Scientific). Thermal cycling parameters were: 30 sec, 98°C; then 35 cycles of: 5 sec, 98°C; 5 sec, 60°C; 15 sec, 72°C; followed by 1 min, 72°C. The diagnostic amplified products were 168 bp (exon 1–2) and 621 bp (exon 1–4).

Expression of porcine GAPDH mRNA was detected by RT-PCR, as above, using primers GAF (5′ TCCCACGGCACAGTCAA 3′) and GAR (5′ GCAGGTCAGGTCCACAA 3′). Thermal cycling parameters were: 2 min, 95°C; then 38 cycles of: 30 sec, 95°C; 30 sec, 60°C; 1 min, 72°C; followed by 5 min, 72°C. The diagnostic amplified product was 575 bp.

### Somatic cell nuclear transfer and embryo transfer

SCNT was performed using *in vitro* matured oocytes as previously described [7]. Cumulus-oocyte complexes (COCs) were aspirated from abattoir ovaries. COCs displaying >3 layers of compacted cumulus cells were selected and cultured in NCSU23 medium supplemented with 0.6 mM cysteine, 10 ng/ml epidermal growth factor, 10% (v/v) porcine follicular fluid, 75 µg/ml potassium penicillin G, 50 µg/ml streptomycin sulphate, 10 IU/ml equine chorionic gonadotropin (eCG; Intervet,) and 10 IU/ml human chorionic gonadotropin (hCG; Intervet). For the first 22 h, COCs were cultured in maturation medium with eCG and hCG, followed by 20 h culture without these hormones in a humidified atmosphere of 5% CO_2_ and 95% air at 38.5°C. Matured oocytes were enucleated by a chemically-assisted method [8]. Oocytes were cultured in NCSU23 medium supplemented with 0.1 µg/ml demecolcine, 0.05 M sucrose, and 4 mg/ml BSA for 0.5–1 h and then enucleated by aspirating the first polar body and adjacent cytoplasm using a bevelled pipette in HEPES-TL-PVP containing 0.1 µg/ml demecolcine, 5 µg/ml cytochalasin B, and 10% foetal calf serum. A single donor cell was inserted into the perivitelline space of an enucleated oocyte. Fusion was performed in 280 mM mannitol solution (pH 7.2) containing 0.15 mM MgSO_4_, 0.01% (w/v) PVA, and 0.5 mM HEPES, by applying a single direct current (DC) pulse (200 V/mm, 20 µs) and a prepulse and postpulse alternating current field of 5 V, 1 MHz for 5 s, respectively (LF101; NEPA Gene, Chiba, Japan). After 0.5–1 h culture in NCSU23, reconstructed embryos were placed in an activation solution consisting of 0.3 M mannitol, 50 µM CaCl_2_, 100 µM MgSO_4_, and 0.01% PVA (300 mosmol) and activated by a single DC pulse of 150 V/mm for 100 µs. Activated oocytes were treated with 5 µg/ml CB for 3 h, then cultured in porcine zygote medium until embryo transfer. Embryo transfer was carried out using the method described by Besenfelder et al. [9]. In brief, 6- to 7-mo-old recipient gilts were oestrus synchronised by oral administration of 4 ml altrenogest (Regumate; Janssen Animal Health, Neuss, Germany) for 15 d, followed by injection of 750 U pregnant mare serum gonadotropin (PMSG; Intergonan; Intervet) 24 h later and 750 U hCG (Ovogest; Intervet) 80 h after PMSG administration. Embryo transfer was conducted 1 d later. Recipients were anaesthetised by intravenous injection of 1.2 ml ketamine hydrochloride (Ursotamin; Serumwerk Bernburg, Germany) per 10 kg body weight (bw) and 0.5 ml xylazine (Xylazin; Serumwerk Bernburg) per 10 kg bw. After fixation of the recipients in dorsal recumbence, SCNT embryos, which were loaded into a flexible intravenous catheter (diameter 1.4 mm, length 45 cm; B. Braun Melsungen AG, Melsungen, Germany), were transferred laparoscopically into the right oviduct. Pregnancy was monitored by ultrasound scanning at regular intervals. Cloned piglets were born naturally by spontaneous parturition.

### Southern blot analysis

Samples of *SbfI* (NEB) digested genomic DNA from targeted MSC clones, kidney tissue from foetuses or piglet ear tip samples were electrophoresed, bound to membrane, hybridised, and probe detected with anti-digoxigenin antibody Fab fragments conjugated with alkaline phosphatase (Roche) by standard methods. The 402 bp *bsr* hybridisation probe was generated by PCR using primers Probe F (5′ ATGGCCAAGCCTTTGTCTC 3′) and Probe R (5′ GATTTAGCCCTCCCACACAT 3′) incorporating alkali labile digoxigenin-11-dUTP (Roche). Thermal cycling parameters were: 2 min, 95°C; then 35 cycles of: 30 sec, 95°C; 30 sec, 56°C; 1 min, 72°C; followed by 5 min, 72°C.

### Cre transduction

Cre protein was produced *in vitro* with the vector pTriEx-HTNC (Addgene plasmid 13763) according to the method described by Peitz *et al.* [10] and Münst *et al*. [11]. 4×10^4^ cells were seeded in a 24 well dish and cultured with 5 µM purified Cre recombinase in culture medium containing 0.5% serum for 16 hours. The medium was then replaced with standard medium and culture continued.

### Cryosections

To collect foetal tissue samples, the sow was first sedated by intramuscular administration of ketamine/azaperone (Intervet), then killed by intravenous injection of T61 (Intervet). Tissue samples from bladder, brain, colon, heart, liver, lung, skin, pancreas and spleen were embedded in OCT embedding compound, frozen on dry ice and stored at −80°C. 5 µm sections were cut using a cryotome (Thermo Fisher Scientific) and fluorescence detected by fluorescence microscopy (Zeiss).

### Whole animal Tomato fluorescence

Tomato fluorescence was revealed in foetuses and piglets using a hand held flashlight with excitation light source UFP-MDS-G2/B/HB, photographs were taken using camera filter FS/CEF-4R2 (Biological Laboratory Equipment Maintenance And Service Ltd., Hungary).

## Results

### Characterisation of the porcine ROSA26 locus

The mouse *Rosa26* locus is widely used as a permissive site for targeted placement of transgenes [12], [13], with no detectable effect on animal viability or fertility. *ROSA26* homologues have also been identified in rat [14] and human [15]. We identified and cloned a highly conserved genomic region on porcine chromosome 13 (NW_003611693∶29648-30716) that shares homology with the promoter and exon 1 regions of mouse (85%), rat (86%) and human (91%) *ROSA26* (Fig. S1A). We then used 3′RACE (rapid amplification of cDNA ends) to identify exons 2 to 4 of porcine *ROSA26* (GenBank Acc.No KF768776). Alignment of the porcine *ROSA26* cDNA sequence with the porcine genomic sequence (NW_003611693) indicated that exon 2 has a size of 112 bp, exon 3 is 118 bp and exon 4 is 480 bp (Fig. S1B). As in mouse and human, porcine *ROSA26* shares a bidirectional promoter with a neighbouring gene *SETD5*. The 3′ *ROSA26* cDNA sequence also overlaps the 3′ region of an adjacent gene *THUMPD3*. Thus the porcine *ROSA26* lies between the *THUMPD3* and *SETD5* genes, as in mouse and human. RT-PCR analysis, using primers located in porcine *ROSA26* exon 1 and 2, and also exon 1 and 4, showed similar levels of expression in all porcine tissues examined (Fig. S1C). All data are thus consistent with identification of porcine *ROSA26.*

### Targeting the porcine *ROSA26* locus

**Figure 1:**
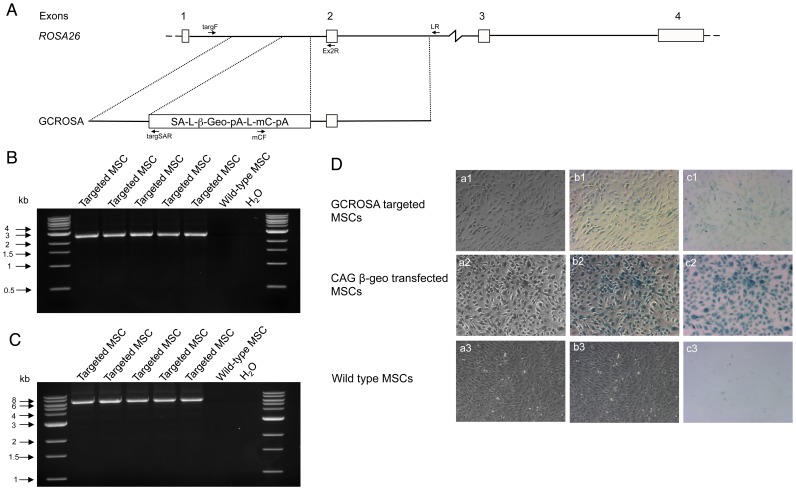
Porcine *ROSA26* gene targeting using GCROSA construct. (A) Top. Porcine *ROSA26* gene. Exon numbers are indicated. Below. GCROSA gene targeting vector. PCR primers used to identify targeted MSC clones are indicated. (B) 5′ junction PCR of five representative GCROSA targeted MSC. Predicted amplified fragment size is 2630 bp. (C) 3′ junction PCR of five representative GCROSA targeted MSC. Predicted amplified fragment size is 6331 bp. (D) lacZ staining of reporter constructs. a1–a3, black and white channel. b1–b3, non-filtered colour channel. c1–c3, colour channel with grey filter. Top panels, GCROSA targeted MSCs; middle panels, MSCs transfected with CAG promoter directed β-geo construct; bottom panels, wild type MSCs.

We placed a β-Geo, mCherry construct into *ROSA26* intron 1 at a site equivalent to that frequently used in mouse *Rosa26*, under the control of the endogenous porcine *ROSA26* promoter (Fig. 1A). Gene targeting using primary bone marrow mesenchymal stem cells (MSC) from a German landrace male pig, resulted in 24 of 50 (48%) cell clones identified as correctly targeted by 5′ and 3′ junction PCR and sequence analysis (Figs. 1B,C). The porcine *ROSA26* locus thus supported conventional gene targeting with very high efficiency. Expression of lacZ directed by the endogenous *ROSA26* promoter was however weak (Fig. 1D), consistent with findings with the mouse *Rosa26* promoter [16].

### Generation of dual fluorescent reporter pigs

**Figure 2:**
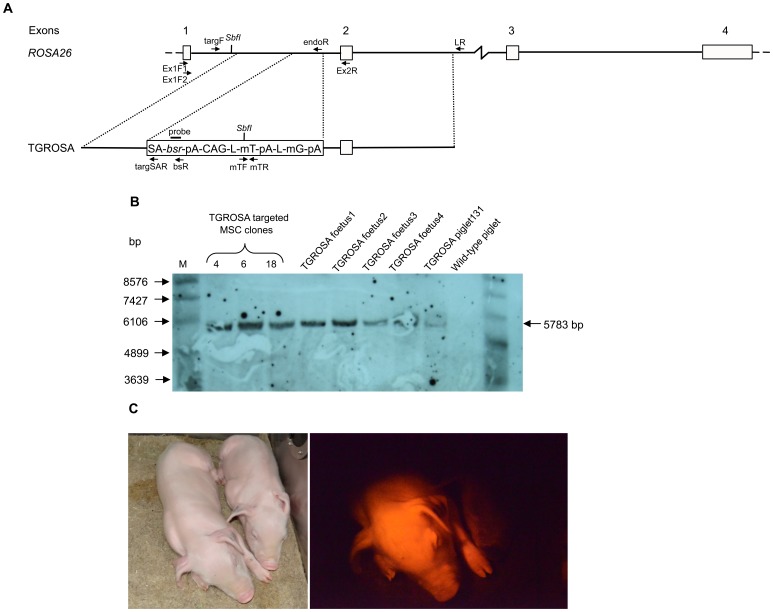
Porcine *ROSA26* gene targeting using TGROSA construct. (A) Porcine *ROSA26* gene targeting scheme. Top. Porcine *ROSA26* gene. Exon numbers are indicated. Below. TGROSA gene targeting vector. PCR and RT-PCR primers used to identify targeted cell cones and detect mRNAs are indicated. *SbfI* restriction sites and the hybridisation probe used for Southern analysis are also shown. (B) Southern blot analysis of TGROSA targeted porcine *ROSA26*. Lanes show *SbfI* digested genomic DNA from three TGROSA targeted MSC clones 4, 6 and 18, four foetuses derived by nuclear transfer, newborn piglet 131 and a wild-type piglet as indicated. The 5783 bp diagnostic fragment detected by the *bsr* probe is shown. (C) Nuclear transfer derived TGROSA piglet (left) and wild-type piglet (right).

**Figure 3:**
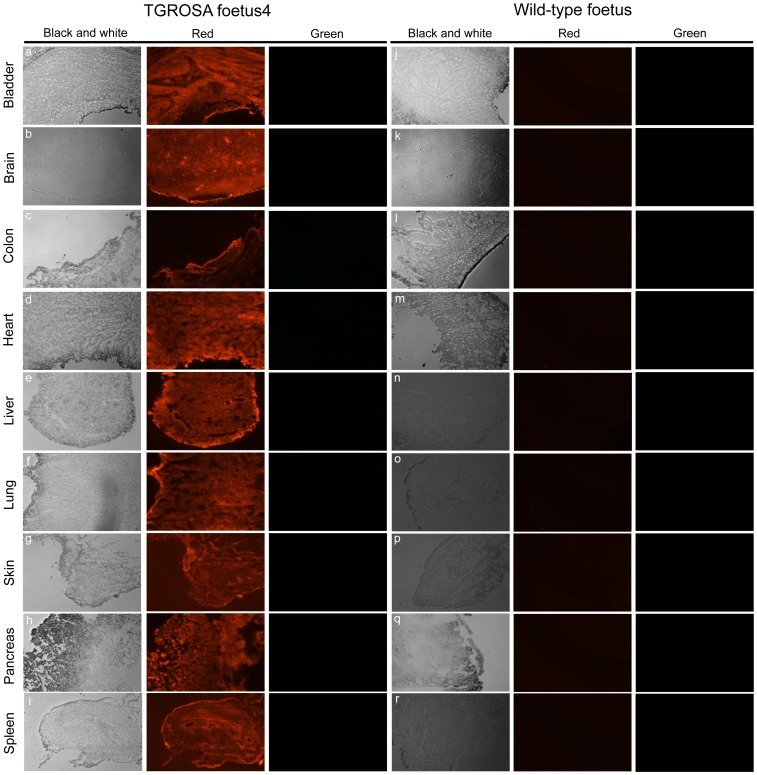
Fluorescence microscopy of tissue cryosections. Panels a-i, organs from a nuclear transfer derived foetus as indicated. Panels j-r, organs from a wild-type foetus. Each section is visualised through black and white, red (excitation = 554 nm, emission = 581 nm) and green (excitation = 489 nm, emission = 509 nm) channels as indicated.

We constructed a second promoter trap vector, TGROSA, to place an mTomato, mGFP dual reporter cassette driven by the constitutive CAG promoter [4] into the same site in intron 1 (Fig. 2A). Gene targeted cell clones were generated using primary adipose MSCs from a German landrace male pig and identified by 5′ and 3′ junction PCR and sequence analysis (Fig. S2A,B). Figure 2B shows detection of a diagnostic fragment by Southern blot analysis. The endogenous *ROSA26* locus was also amplified to determine whether targeting had occurred at one or both alleles (Fig. S2C). Again the targeting efficiency was high, with 16 of 38 (42%) cell clones identified as correctly targeted, all at one allele. Three targeted cell clones (4, 6 and 18, Figure 2B) were pooled and used for nuclear transfer and pregnancies established. One pregnant sow was sacrificed and four foetuses explanted for analysis, all showed bright Tomato fluorescence (Fig. S2D). Other pregnancies were allowed to continue to birth. Two normal healthy piglets were identified by mTomato fluorescence (e.g. Figure 2C) and confirmed by PCR amplification across the 5′ and 3′ junction regions (Fig. S2E). When mature these will be mated and used to found a reporter pig line. One other normal healthy piglet (131) was unfortunately killed by the sow. PCR and Southern analysis of foetuses and piglets confirmed *ROSA26* targeting identical to the cell clones (Figure 2B, Fig. S2A,B,C). The pattern of reporter gene expression was analysed in the dead piglet 131. RNA samples from spleen, lung, pancreas, kidney, muscle, liver, heart and skin were tested by RT-PCR for the presence of RNA transcribed from the *ROSA26* promoter extending from exon 1 spliced to the blasticidin gene, and also for mTomato RNA transcribed from the CAG promoter. Both RNA species were detected in all samples (Fig. S3A,B,C). Cryosections were prepared from major organs including bladder, brain, colon, heart, liver, lung, skin, pancreas and spleen from foetus 4. Tomato fluorescence was evident in all organs examined (Fig. 3).

### Functional analysis of dual reporter

**Figure 4:**
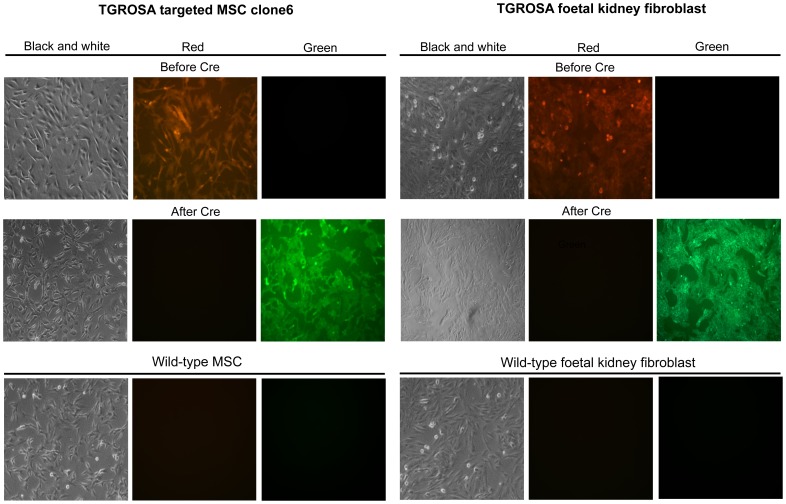
Cre-induced loss of Tomato and activation of GFP fluorescence in TGROSA targeted MSC cell clones and kidney fibroblasts from a nuclear transfer derived foetus. Fluorescence before, and eight days after Cre transduction as indicated. Wild-type MSCs and foetal kidney fibroblasts are shown.

To investigate the response of the dual reporter to Cre activity, TGROSA targeted MSC cell clone 6 and kidney fibroblasts prepared from an explanted foetus were transduced with Cre protein. Prior to Cre transduction GFP fluorescence was not detected in either cell type (Fig. 4), while strong Tomato fluorescence was evident in both (Fig. 4). After Cre transduction, GFP fluorescence became evident and Tomato fluorescence steadily declined over a period of 8 days until it was absent from almost all cells. This time course was consistent with the relatively long half-life of mTomato protein and was similar to that observed in mice [4].

## Discussion

We have generated pigs using the most successful dual reporter system currently available. Pigs with Cre-inducible single reporter genes have been reported previously [17], [18], but it is widely recognised that dual reporters are more useful and reliable indicators of Cre recombination, because one reporter gene is always active in any individual cell providing an internal control.

We provide a full and comprehensive characterisation of the porcine *ROSA26* locus. Our results accord with the partial description published recently [18], although we identified four rather than two exons. This suggests that porcine *ROSA26* expresses multiple transcript variants, as found in other species [19].

Our results also demonstrate that the porcine *ROSA26* locus can be readily targeted in primary somatic cells with very high efficiency using traditional targeting vectors, and that it supports ubiquitous expression of an inserted transgene, consistent with results from mouse, rat, and human. In other work we also obtained efficient targeting (28% of cell clones analysed) when placing an atherosclerosis marker gene under the control of a tissue-specific promoter at the same site. These findings contrast with those recently described by Li *et al.*, who reported poor targeting efficiency into porcine *ROSA26* using a conventional vector [18], which could be due to the different location they used within *ROSA26* intron 1. Efficient gene targeting is an important factor if, as in mice, porcine *ROSA26* is to be used a ‘general purpose’ permissive locus for transgene placement. Reliable ubiquitous or tightly controlled transgene expression, free of position effect variation or silencing, is a basic requirement for many new biomedical applications where genetically modified pigs are playing an increasingly important role [20].

We have already established conditional gene targeting in pigs [2] and are currently generating Cre-driver lines. The dual Cre reporter animals described here will be used to establish a reporter line to characterise and monitor Cre-driver pigs designed to express Cre recombinase in specific cell types, at defined time points, or in response to drug induction, as widely used in mice [21]. The Cre reporter pig line can also be used to directly assess the pattern of Cre expression administered locally, for example by *in vivo* viral transduction, DNA transfection, or protein transduction [22], [23]. We are confident that it will be an important and useful resource that will enable sophisticated techniques of genetic modification and precise control of gene expression in pigs.

## Supporting Information

Figure S1 **Identification and expression of the porcine** ***ROSA26*** **locus.** (A) DNA sequence alignment of the promoter region and exon1 of *ROSA26* in mouse, rat, pig and human. The porcine sequence shown is located on chromosome 13 (NCBI *Sus Scrofa* 10.2 porcine genome NW_003611693∶29648–30716). The black line indicates porcine *ROSA26* exon1. The mouse, rat and human *ROSA26* sequences shown are located on chromosome 6 (AC_000028), chromosome 4 (NC_005103) and chromosome 3 (NC_000003) respectively. (B) Porcine *ROSA26* cDNA with the four exons indicated by different colours. (C) Expression of porcine *ROSA26* in different adult tissues detected by RT-PCR. The primers anneal in exon 1 and exon 2 and amplify a correctly spliced product of 168 bp (upper). The primers anneal in exon 1 and exon 4 and amplify a correctly spliced product of 621 bp (middle). *GAPDH* expression was used as a control for RNA quality (lower).
(PDF)

Figure S2 **PCR screening of TGROSA targeted MSC clones 4, 6 and 18, four nuclear transfer derived foetuses, newborn TGROSA piglet 131 and two normal healthy piglets.** (A) PCR detection of TGROSA targeted 5′ terminal region. Amplified fragment size: 2630 bp. (B) PCR detection of TGROSA targeted 3′ terminal region. Amplified fragment size: 7868 bp. (C) PCR detection of wild type *ROSA26* allele. Amplified fragment size: 3206 bp. (D) TGROSA foetuses and wild type foetus (mTomato fluorescence above and bright light below). (E) 5′ junction PCR (left) and 3′ junction PCR (right) for two normal healthy piglets. Amplified fragment sizes are 2630 bp and 7868 bp respectively.
(PDF)

Figure S3 **RT-PCR screening of newborn TGROSA piglet 131.** (A) RT-PCR detection of targeted *ROSA26* RNA from exon1 spliced to the blasticidin selectable gene (*bsr*) in different tissues derived from TGROSA piglet 131. Amplified fragment size: 500 bp. (B) RT-PCR detection of mTomato RNA in different tissues. Amplified fragment size: 686 bp. (C) RT-PCR for housekeeping gene GAPDH in different tissues derived from TGROSA piglet 131. Amplified fragment size: 575 bp. In each case a wild-type piglet and H_2_O controls are indicated.
(PDF)
