# Supplementary material for: Dual Fluorescent Reporter Pig for Cre Recombination: Transgene Placement at the ROSA26 Locus
Source: PLoS One. 2014 Jul 15;9(7):e102455. doi: 10.1371/journal.pone.0102455 (PMC4099177; doi:10.1371/journal.pone.0102455)
Supplement: Figure S1 — Identification and expression of the porcine ROSA26 locus. (A) DNA sequence alignment of the promoter region and exon1 of ROSA26 in mouse, rat, pig and human. The porcine sequence shown is located on chromosome 13 (NCBI Sus Scrofa 10.2 porcine genome NW_003611693∶29648–30716). The black line indicates porcine ROSA26 exon1. The mouse, rat and human ROSA26 sequences shown are located on chromosome 6 (AC_000028), chromosome 4 (NC_005103) and chromosome 3 (NC_000003) respectively. (B) Porcine ROSA26 cDNA with the four exons indicated by different colours. (C) Expression of porcine ROSA26 in different adult tissues detected by RT-PCR. The primers anneal in exon 1 and exon 2 and amplify a correctly spliced product of 168 bp (upper). The primers anneal in exon 1 and exon 4 and amplify a correctly spliced product of 621 bp (middle). GAPDH expression was used as a control for RNA quality (lower). (PDF) [file pone.0102455.s001.pdf]

A

```
Mouse           TGGGCCCCCACGAGCGACCAGAGTTGTC----ACAAGGCCGCAAGAACAGGGGAGGTG-G 55
Rat             TGGGCCCCCACGAGCGATCAGAGTTGTC----ACAAGGCCGCAAGAACAGGGGTGGAG-G 55
Pig             --GGGCCCCA-GAGCGACCAGAGTTGTC----ACAAGGCCGCGCGAACGGGGGTGGGG-G 52
Human           --GGCCCCCA-GAGCGATCAGAGTTGTCTGTCACAAGGCCGCGAGAACGGGGGTAGGGAG 57
                  ** ***** ****** **********    **********  **** ****  * * *

Mouse           --GGGGCTCAGGGACAG-AAAAAAAAGTATG----TGTATTTTGAG--AGCAGGGTTGGG 106
Rat             TGGGGGCTCAGGGACAG-AAAAAAAAGTATG----TGTATTTTGAG--AGCAGGATTGAG 108
Pig             T-GGGGTTTGGGGAGGGGAAAAAAAAGTGTGC-TGTGTATTTTGAG--GAGGGCGGCGAG 108
Human           TGGGGGATCGGGGAGAG--AAAAAAAGTATGCCTGTGTATTTCGAGCGGAGGGCAGCAAG 115
                  **** *  ****  *  ********* **    ******* ***      *      *

Mouse           AGGCCTCTCCTGAAA-AGGGTATAAACGTGGAGTAGGCAATACCCAGGCAAAAAGGGGAG 165
Rat             AGGCCTCTCGTGAAA-AGGATATAAACGTGAAGTAGGCAGTACCCAG--AAAAAGAGGAG 165
Pig             AGGCCTATTCTCAAGTAAAAGGTAAACGTGGAGTAGGCAGTTCACAG--GAAAAGGGGTG 166
Human           AGGCCTCTCCTCAAGGGAAAGGTAAACGTGGAGTAGGCAGTTCCCAG--GAAAGGGGGTG 173
                ****** *  * **          ******** ******** * * ***   *** * ** *

Mouse           ACCAGA-GTAGGGGGAGGGGAAGAGTCCTGACCCAGGGAAGACATTAAAAAGGTAGTGGG 224
Rat             ACTAGA-GTAGGGGGAGGGGAAGAGTCCTGACCCAGGGAAGACATTAAAAAGGTAGTGGG 224
Pig             AAGAGGCGTGGGGGGAGGGGAAACGTCCTGACCCAGGAAAGACATGAAAAGGGTAGTGGG 226
Human           AAGAGGCGTTGGGGGAGGGGAAGCGTCCTGACCCAGGAAAAACATGAAAGGGGGAGTTGG 233
                *  **  ** ************  ************* ** **** ***   ** *** **

Mouse           GTCGACTAGATGAAGGAGAG--CCTTTCTCTCTGGGCAAGAGCGGTG---CAATGGTGTG 279
Rat             GTCGACTAGATTAAGGGGAG--CCTTTCTCCCTGGGCAAGAGGGGTG---CAGTGGTGTG 279
Pig             GTCGACTAGATTAAGGAGGGGGCCTCTCCGCCTGGGAAAGAGGGGTA---CAGTGGTGTG 283
Human           GTCGCCTAGATTAGAGGGGGA-TCTCTCTCCCTGGGAAAATGGGGTGTTGCAACGGTGTG 292
                **** ****** *  * * *   ** **   ***** **  * ***     **  ******

Mouse           TA-AAGGTAGCTGAGAAGACGAAAAGGGCAAGCATCTTCCTGCTACCAGGCTGGGGAGGC 338
Rat             TA-AAGGTAGCCGAGAAGATGAAAAGGGCAAGCATCTTCCTGCTACCAGCCTGGGGAGGC 338
Pig             GG-GGGGCGA--GGGGGGATGGAAGGGGCAGCATCCTCCTGCTGAGAGCCGGGGGAGGG 340
Human           TGCAAGGCGGCGAGGGGGGTGAGAAGTGGCAGCATCCTCCT---AAGAGCTTGGGGAGGG 349
                     **        *  *  *  *** *  ****** ****       **   *******

Mouse           CCAGGCCCACGACCCCGAGGAGAGGGAACGCAGGGAGACTGAGG---TGACCCTTCTTTC 395
Rat             CCAGGCCCACGACACCGAGGAGAGGGAACGCTGGGAGACTGAGG---TGACCCTTCTT-C 394
Pig             CCAGGCCCACGTCCC----GAGAGCAAGCGCGAGGAGACGGAGGAGGTGACCCTTCCCTC 396
Human           CCAGGCCCACGACCC-AAGGAGAGCGAGCGCGGGGAGACGGAGGAGGTGACCCTTCCCTC 408
                *********** * *    *****  * ***  ****** ****   *********   *

Mouse           CCCCGGGGCCCGGTCGTGTGGTTCGGTGTCTCTTTTCTGTTGGACCCTTACCTTGACCCA 455
Rat             CCCCGGGGCCCGGTCGTGTGGTTCGGTGTCTCTTTTCTGTTGGACCCTTACCTTGACCCA 454
Pig             CCCCGGGGCCCGGTGGTGAGGGGAGGTCTCTCTTTTCTGTCGCACCCTTACCTTGTCCCA 456
Human           CCCTGGGGCCCGATCGTGAGGTTCGG--TCTCTTTTCTGTCGGACCCTTACCTTGTCCCA 466
                *** ******** * *** **    **  ************ * ************ ****

Mouse           GGCGCTGCCGGGGCCTGGGCCCGGGCTGCGGCGCACGGCACTCCCGGGAGGCAGCGAGAC 515
Rat             GGCGCTGCCGGGGCCTGGGCCCGGGCTGCGGCGCACGGCACTCCCGGGAGGCAGCGAGAC 514
Pig             G-----------GCCTGGGCCCGGGCTGCGGCGCACGGCACTCCCGGTAGGCAGCAGGAC 505
Human           GGCGCTGCCGGGGCCTGGGCCCGGGCTGCGGCGCACGGCACTCCCGGGAGGCGGCAGGAC 526
                *           *********************************** **** **  ***

Mouse           TCGAGTTAGGCCCAACGCGGCGCCACGGCGTTTCCTGGCCGGGAATGGCCCGTACCCGTG 575
Rat             TCGAGTTAGGCCCAACGCGGCGCCACGGCGTTTCCTGGCCGGGAATGGCCCGTACCCGTG 574
Pig             TCGAGTTAGGCCCAGCGCGGCGCCACGGCGTTTCCTGGCCGGGAATGGCCCGTGCCCGTG 565
Human           TCGAGTTAGGCCCAACGCGGCGCCACGGCGTTTCCTGGCCGGGAATGGCCCGTACCCGTG 586
                ************** ************************************** ******

Mouse           AGGTGGGGGTGGGGGGCAGAAAAGGCGGAGCGAGCCCGAGGCGGGGAGGGGG---AGGGC 632
Rat             AGGTGGGGGTGGGGGGCAGAAAAGGCGGAGCGAGCCCAAGGCGGGGAGGGGG---AGGGC 631
Pig             AGGTGGGGGTGGGGGGCA-AAAAGGCGGAGCGAGCCAAAGGCGGTGAGGGGGG--AGGGC 622
Human           AGGTGGGGGTGGGGGGCA-GAAAGGCGGAGCGAGCCAAAGGCGGGGAGGGGGGGCAGGGC 645
                ******************  ****************  ****** *******   *****

Mouse           CAGGGGCGGAGGGGG---CCGGCACTACTGTGTTGGCGGACTGGCGGGACTAGGGCTGCG 689
Rat             CAGGGGCGGAGGGGGG--CCGGCACTACTGTGTTGGCGGACTGGCGGGACTAGGGCTGCG 689
Pig             CAGGGAAGGAGGGGGGGGCCGGCACTACTGTGTTGGCGGACTGGCGGGACTGGGGCTGCG 682
Human           CAGGGAAAGAGGGGGG--CCGGCACTACTGTGTTGGCGGACTGGCGGGACTGGGGCTGCG 703
                *****   *******   ********************************* ********

Mouse           TGAGTCTCTGAGCGCAGGCGGGCGGCGGCCGCCCCTCCCCCGGC------GGCGGCAGCG 743
Rat             TGAGTCTCTGAGCGCAGGCGGGCGGCGGCCGCCCCTCCCCCGGC------GGCGGCAGCG 743
Pig             TGAGTCTCTGAGCGCAGGCGGGCGGCGGCCGCCCCTCCCCCGGC------GGCGGCGGCG 736
Human           TGAGTCTCTGAGCGCAGGCGGGCGGCGGCCGCCCCTCCCCCGGCAGCGGCGGCGGCGGCG 763
                ********************************************      ****** ***

Mouse           GCGGCAGCGGCGGC------AGCTCACTCAGCCCGCTGCCCGAGCGGAAACGCCACTGAC 797
Rat             GCGGCAGCGGCGGC------AGCTCACTCAGCCCGCTGCCCGAGCGGAAACGCCACTGAC 797
Pig             GCGGCGGCGGCGGCGGCAGCAGCTCACTCAGCCCGCTGCCCGAGCGGAAACGCCACTGAC 796
Human           GCGGCGGCGGCGGCGGCGGCAGCTCACTCAGCCCGCTGCCCGAGCGGAAACGCCACTGAC 823
                ***** ********      ****************************************

Mouse           CGCACGGGGATTCCCAGTGCCGGCGCCAGGGGCACGCGGACACGCCCCCTCCCGCCGCG 857
Rat             CGCACGGGGATTCCCAGTGCCGGCGCCAGGGGCACGCGGACACGCCCCCTCCCGCCGCG 857
Pig             CGCACGGGGATTCCCAGCGCCGGCGCCAGGGGCACCCGGACACGCCCCCTCCCGCCGCG 856
Human           CGCACGGGGATTCCCAGCGCCGGCGCCAGGGGCACCCGGACACGCCCCCTCCCGCCGCG 883
                ***************** ***************** ***********************

Mouse           CCATTGGCCTCTCCGCCCACCGCCCCACACTTATTGGCCGGTGCGCCGCCAATCAGCGGA 917
Rat             CCATTGGCCTCTCCGCCCACCGCCCCACACTTATTGGCCAGCTCCCCGCCAATCAGCGGA 917
Pig             CCATTGGCCCCTCCGCCCACCGTCTCGCACCCATTGGCCAGCTCCCCGCCAATCAGCGGA 916
Human           CCATTGGCCTCTCCGCCCACCGCCCCGCACCCATTGGCCCACTCGCCGCCAATCAGCGGA 943
                ********* ************ * * ***  *******    * ***************

Mouse           GGCTGCCGGGGCCGCCTAAAGAAGAGGCTGTGCTTTGGGGCTCCGGCTCCTCAGAGAGCC 977
Rat             GGCTGCCGGGGCCGCCTAAAGAAGAGGCTGTGCTCTGGGGCTCCGGCTCCTCAGAGAGCC 977
Pig             AGCCGCCGGGGCCGCCTAGAGAAGAGGCTGTGCTCTGGGGCTCCGGCTCCTCAGAGAGCC 976
Human           AGCCGCCGGGGCCGCCTAGAGAAGAGGCTGTGCTTCGGCGCTCCGGCTCCTCAGAGAGCC 1003
                 ** ************** ***************  ** ********************

Mouse           TCGGCTAGGTAGGGGATCGGGACTCTGGCGGGAGGGCGGCT-TGGTGCGTTTGCGGGG-A 1035
Rat             TCGGCTAGGTAGGGGATCGGGACTCTGGCGGGAGGGTGGCT-TGGCGCGTTTGCGGGG-G 1035
Pig             TCGGCTAGGTAGGGGAGCGGGACTCTGGTTTGGGGGAGGGC-CGGCGGTTTGGCGGGGGA 1035
Human           TCGGCTAGGTAGGGGAGCGGAACTCTGGTGGGAGGGGAGGTGCGGTACACTGG-GGGG-A 1061
                **************** *** *******    * *** *      **     * * ****

Mouse           TGGGCGGCCGCGGCAGGCCCTCCGAGCGTG-GTGG 1069
Rat             CGGGCGGCCGCGGTAGGCCCTCCAAGGACG-GTGG 1069
Pig             TGGGTGCTTGAGGTGGTCTGACCGGTAGCG-GGGG 1069
Human           TGGGTGGCTAGGG-GGGCCGTCTGGTGGCTTGCGG 1095
                 *** *     **  * *   *         * **
```

B

GGAAGCCGCCGGGGCCGCCTAGAGAAGAGGCTGTGCTCTGGGGCTCCGGCTCCTCAGAGA
GCCTCGGCTAGTTTTAATTTCTAGTATGGTAAAATACTGGTAAACAAAGCATTTGGGACCCTC
AGCTTTTAATAATGTGAAGATATCCTGAGACCAAGAAGTTGGAGGAAGCTGCTAAGCATACC
AATGGATTATTATCGCCAGCAATATGGTAACAGTTAGACCATTCTGTAGCCCCCTAAAAGACA
AGAGAATATATTAAAAGAGAAGTAACAAACTGCAAAACAGAAAAGATTAAAGGGCCACACTT
GCATCATATGAAGAACTCTAGAGGTTGAATTGGAGCTGTAGCGGCCAGCCTATGTTACAGCC
ACAGCAACCTGGGATCCAAGCTCAATCTGTGACCTATAACACAGATCATGGCAATGCTGGAT
CCTTAACCCACTGAGCGAGGCCAGGGATCAAACCTACATCCTCATAGATCCTAGTCGGGCTC
GTTAACTGCTGAGCCACAAAGGGAACTCCCTTATTTATTTGCATTTTATTTTTGTCTTTTTAGG
GCTGCATCCACAGCCTGTGTAAGTTCCCAGGCTAGGGGCTGAAGCGGAGCTATAGCTGTCAG
CCTACACCACAGCCACAGCAGTGCCAGATCCTAGTGGTGTCTGTGACCTACACCACAACTCA
CAGCAATGCCGGATCCTTAACCACTGAGCCAGGACAGGGATTAAACACACATCCTCATGGAT
ACTAGTTGGGGTTCCTTATAGCTGAAGTCATCATG

C

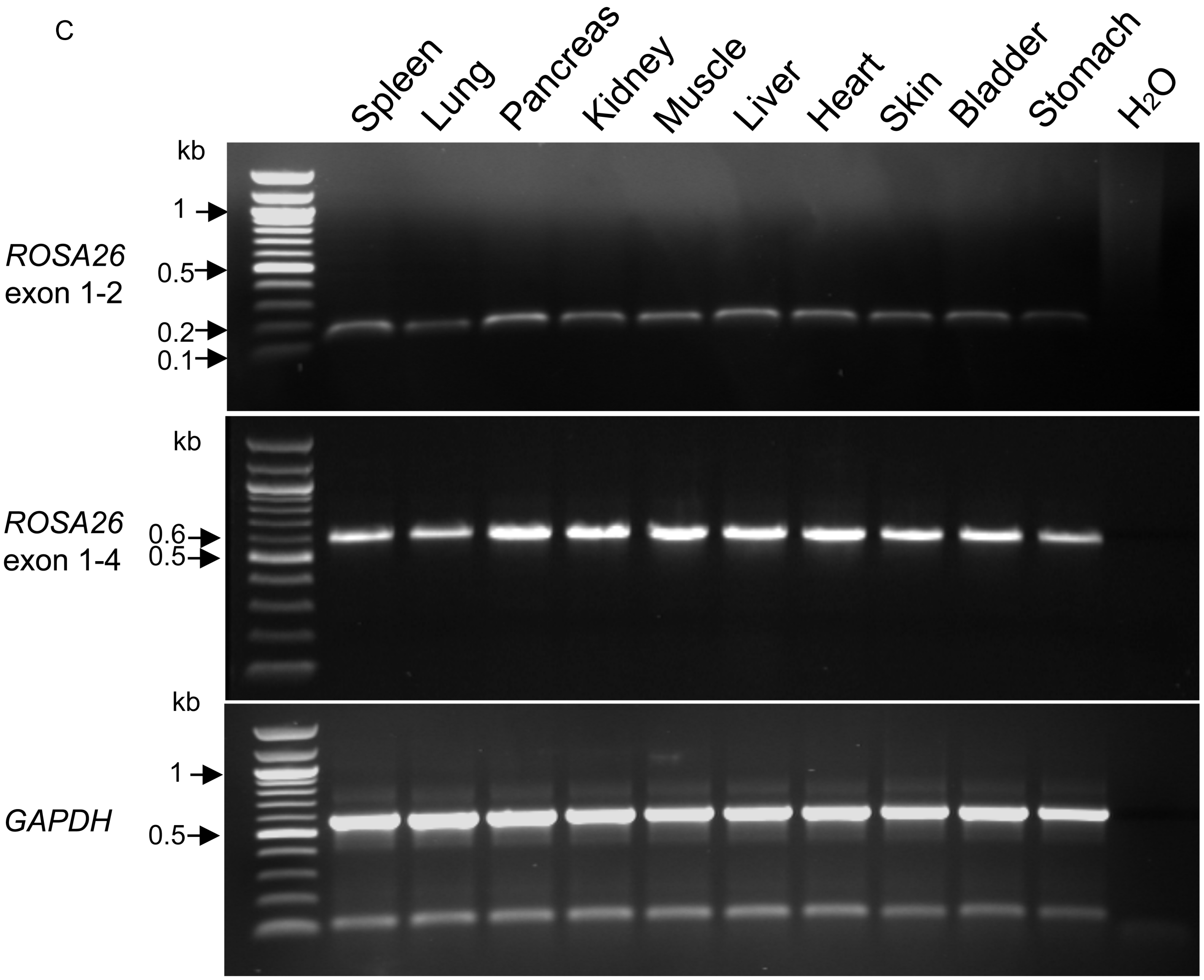

Figure S1. Identification and expression of the porcine *ROSA26* locus. (A) DNA sequence alignment of the promoter region and exon1 of *ROSA26* in mouse, rat, pig and human. The porcine sequence shown is located on chromosome 13 (NCBI Sus Scrofa 10.2 porcine genome NW_003611693: 29648-30716). The black line indicates porcine *ROSA26* exon1. The mouse, rat and human *ROSA26* sequences shown are located on chromosome 6 (AC_000028), chromosome 4 (NC_005103) and chromosome 3 (NC_000003) respectively. (B) Porcine *ROSA26* cDNA with the four exons indicated by different colours. (C) Expression of porcine *ROSA26* in different adult tissues detected by RT-PCR. The primers anneal in exon 1 and exon 2 and amplify a correctly spliced product of 168 bp (Above). The primers anneal in exon 1 and exon 4 and amplify a correctly spliced product of 621 bp (Middle). *GAPDH* expression was used as a control for RNA quality (Below).
