# Supplementary material for: Dual Fluorescent Reporter Pig for Cre Recombination: Transgene Placement at the ROSA26 Locus
Source: PLoS One. 2014 Jul 15;9(7):e102455. doi: 10.1371/journal.pone.0102455 (PMC4099177; doi:10.1371/journal.pone.0102455)
Supplement: Figure S2 — PCR screening of TGROSA targeted MSC clones 4, 6 and 18, four nuclear transfer derived foetuses, newborn TGROSA piglet 131 and two normal healthy piglets. (A) PCR detection of TGROSA targeted 5′ terminal region. Amplified fragment size: 2630 bp. (B) PCR detection of TGROSA targeted 3′ terminal region. Amplified fragment size: 7868 bp. (C) PCR detection of wild type ROSA26 allele. Amplified fragment size: 3206 bp. (D) TGROSA foetuses and wild type foetus (mTomato fluorescence above and bright light below). (E) 5′ junction PCR (left) and 3′ junction PCR (right) for two normal healthy piglets. Amplified fragment sizes are 2630 bp and 7868 bp respectively. (PDF) [file pone.0102455.s002.pdf]

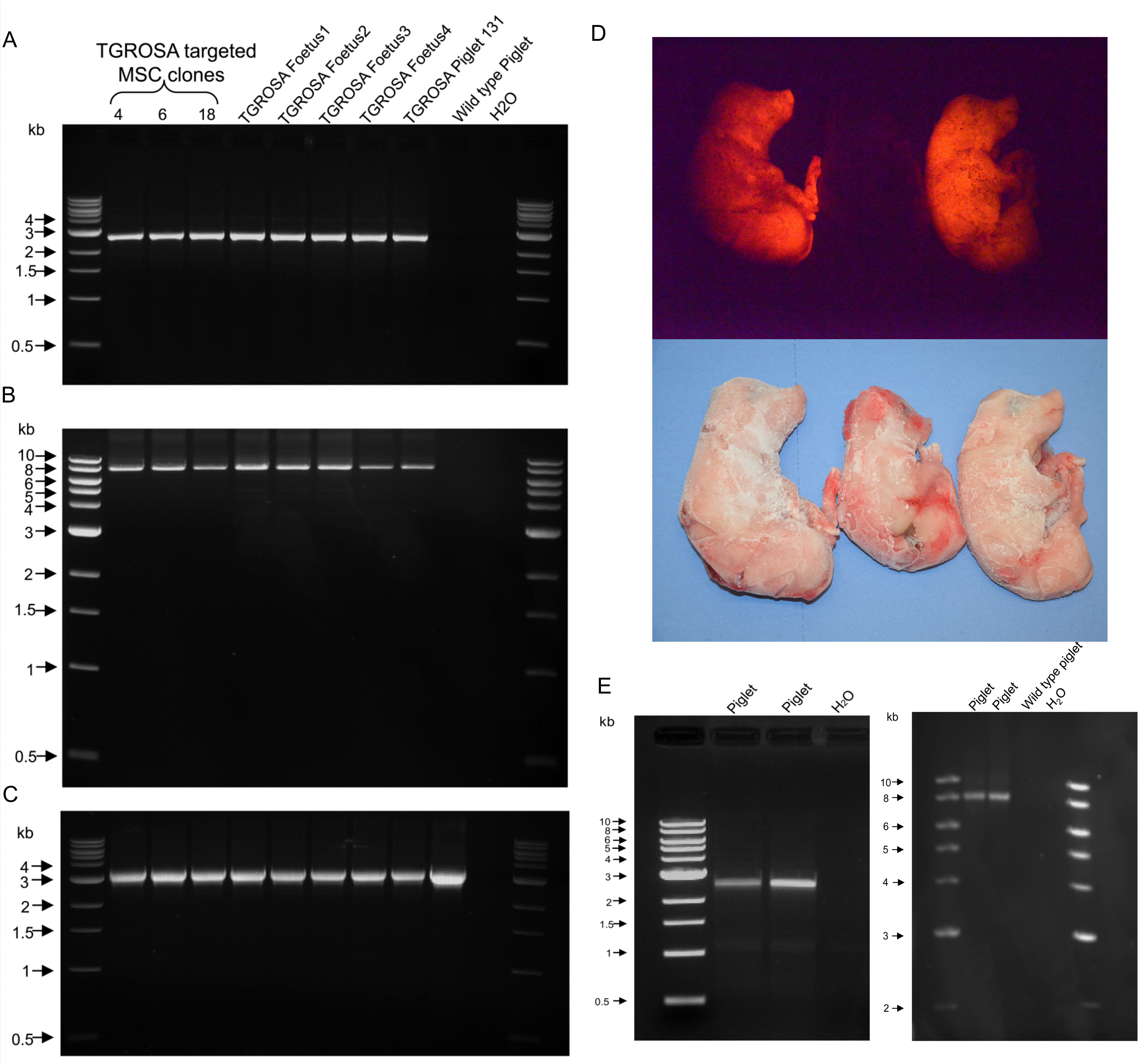

Figure S2. PCR screening of TGROSA targeted MSC clones 4, 6 and 18, four nuclear transfer derived foetuses, newborn TGROSA piglet 131 and two normal healthy piglets. (A) PCR detection of TGROSA targeted 5’ terminal region. Amplified fragment size: 2630 bp. (B) PCR detection of TGROSA targeted 3’ terminal region. Amplified fragment size: 7868 bp. (C) PCR detection of wild type *ROSA26* allele. Amplified fragment size: 3206 bp. (D) TGROSA foetuses and wild type foetus (mTomato fluorescence above and bright light below). (E) 5' junction PCR (left) and 3' junction PCR (right) for two normal healthy piglets. Amplified fragment sizes are 2630 bp and 7868 bp respectively.
