# Supplementary material for: Dual Fluorescent Reporter Pig for Cre Recombination: Transgene Placement at the ROSA26 Locus
Source: PLoS One. 2014 Jul 15;9(7):e102455. doi: 10.1371/journal.pone.0102455 (PMC4099177; doi:10.1371/journal.pone.0102455)
Supplement: Figure S3 — RT-PCR screening of newborn TGROSA piglet 131. (A) RT-PCR detection of targeted ROSA26 RNA from exon1 spliced to the blasticidin selectable gene (bsr) in different tissues derived from TGROSA piglet 131. Amplified fragment size: 500 bp. (B) RT-PCR detection of mTomato RNA in different tissues. Amplified fragment size: 686 bp. (C) RT-PCR for housekeeping gene GAPDH in different tissues derived from TGROSA piglet 131. Amplified fragment size: 575 bp. In each case a wild-type piglet and H2O controls are indicated. (PDF) [file pone.0102455.s003.pdf]

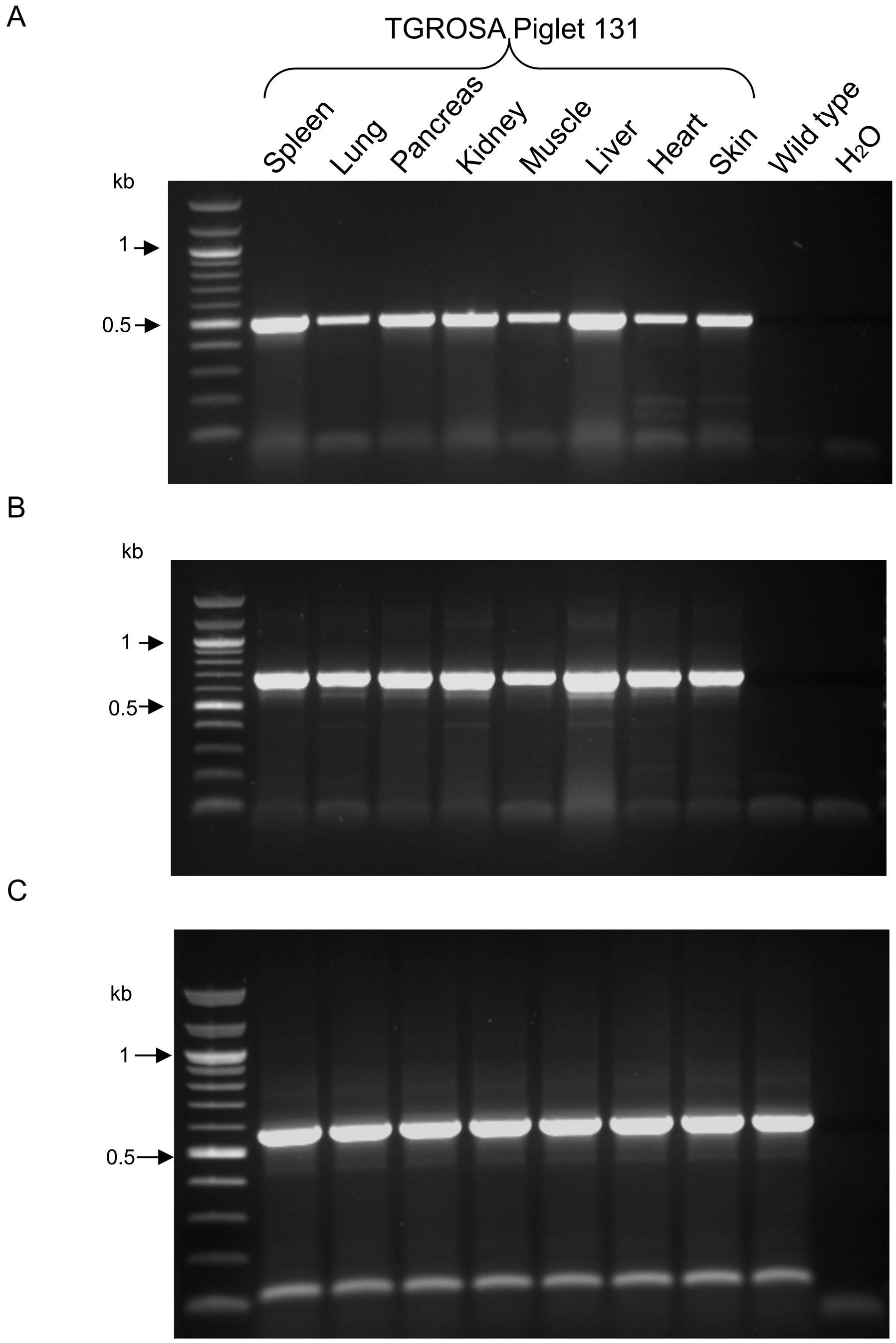

Figure S3. RT-PCR screening of newborn TGROSA piglet 131. (A) RT-PCR detection of targeted *ROSA26* RNA from exon1 spliced to the blasticidin selectable gene (*bsr*) in different tissues derived from TGROSA piglet 131. Amplified fragment size: 500 bp. (B) RT-PCR detection of mTomato RNA in different tissues. Amplified fragment size: 686bp. (C) RT-PCR for housekeeping gene GAPDH in different tissues derived from TGROSA piglet 131. Amplified fragment size: 575 bp. In each case a wild-type piglet and H_2O controls are indicated.
